# Silicon Alleviate Hypoxia Stress by Improving Enzymatic and Non-enzymatic Antioxidants and Regulating Nutrient Uptake in Muscadine Grape (*Muscadinia rotundifolia* Michx.)

**DOI:** 10.3389/fpls.2020.618873

**Published:** 2021-02-10

**Authors:** Zafar Iqbal, Ali Sarkhosh, Rashad Mukhtar Balal, Celina Gómez, Muhammad Zubair, Noshin Ilyas, Naeem Khan, Muhammad Adnan Shahid

**Affiliations:** ^1^Central Laboratories, King Faisal University, Al-Hofuf, Saudi Arabia; ^2^Horticultural Sciences Department, University of Florida, Gainesville, FL, United States; ^3^Department of Horticulture, College of Agriculture, University of Sargodha, Sargodha, Pakistan; ^4^Environmental Horticulture Department, University of Florida, Gainesville, FL, United States; ^5^Department of Botany, Pir Mehr Ali Shah Arid Agriculture University, Rawalpindi, Pakistan; ^6^Agronomy Department, University of Florida, Gainesville, FL, United States; ^7^Department of Agriculture, Nutrition and Food Systems, University of New Hampshire, Durham, NH, United States

**Keywords:** antioxidants, flooding, hypoxia, muscadine grape, nanoparticles, photosynthesis, osmoprotectants

## Abstract

Flooding induces low oxygen (hypoxia) stress to plants, and this scenario is mounting due to hurricanes followed by heavy rains, especially in subtropical regions. Hypoxia stress results in the reduction of green pigments, gas exchange (stomatal conductance and internal CO_2_ concentration), and photosynthetic activity in the plant leaves. In addition, hypoxia stress causes oxidative damage by accelerating lipid peroxidation due to the hyperproduction of reactive oxygen species (ROS) in leaf and root tissues. Furthermore, osmolyte accumulation and antioxidant activity increase, whereas micronutrient uptake decreases under hypoxia stress. Plant physiology and development get severely compromised by hypoxia stress. This investigation was, therefore, aimed at appraising the effects of regular silicon (Si) and Si nanoparticles (SiNPs) to mitigate hypoxia stress in muscadine (*Muscadinia rotundifolia* Michx.) plants. Our results demonstrated that hypoxia stress reduced muscadine plants’ growth by limiting the production of root and shoot dry biomass, whereas the root zone application of both Si and SiNP effectively mitigated oxidative and osmotic cell damage. Compared to Si, SiNP yielded better efficiency by improving the activity of enzymatic antioxidants [including superoxide dismutase (SOD), peroxidase (POD), and catalase (CAT)], non-enzymatic antioxidants [ascorbic acid (AsA) and glutathione contents], and accumulation of organic osmolytes [proline and glycinebetaine (GB)]. SiNP also regulated the nutrient profile of the plants by increasing N, P, K, and Zn contents while limiting Mn and Fe concentration to a less toxic level. A negative correlation between antioxidant activities and lipid peroxidation rates was observed in SiNP-treated plants under hypoxia stress. Conclusively, SiNP-treated plants combat hypoxia more efficiently stress than conventional Si by boosting antioxidant activities, osmoprotectant accumulation, and micronutrient regulation.

## Introduction

Global climatic changes are predicted to cause extreme environmental conditions such as floods, heat waves, and droughts, in all parts of the world. The high occurrence of flooding is causing globally more climate-related disasters to crops, agricultural assets, and infrastructure than any other abiotic factor and has increased by approximately 65% over the last 25 years (Egal, 2019). Damage caused by flooding is difficult to assess, primarily due to the complex nature of its occurrence. It can also vary significantly, depending on the amount, intensity, duration, and spatial distribution of precipitations, all of which make ecosystems vulnerable worldwide (Ruperti et al., 2019).

Flooding results in partial or whole submergence of plants, affecting all growth stages, from seed germination to vegetative and reproductive growths. Flooding causes acute hypoxia, i.e., low oxygen conditions (usually between 1 and 5%). Under hypoxia stress, the cellular and physiological functions of the plants are compromised and negatively affect the developmental stages of the plants. The limited aerobic respiration causes a decrease in energy and limits plant development (Zhou et al., 2020). Plants respond to hypoxia stress at morphological, physiological, biochemical, and molecular levels by modifying their metabolism, gene expression, photosynthesis, and phytohormonal balance.

Although technological advancements and innovations have been made in recent years to meet the growing challenges of sustainable production and food security in a changing climate, there is still a dire need for novel and contemporary approaches for sustainable crop production (Vandermeer et al., 2018). The exploration and exploitation of novel strategies complementing existing conventional approaches have become very crucial for sustainable crop production. In particular, nanotechnology has the potential to provide practical solutions to multiple agriculture-related problems. The use of certain nanomaterials has produced promising results in agricultural research. In agriculture, the novel properties of nanomaterials that can play a role in the crop improvement program, as well as the alleviation of different stresses, are being exploited (Das and Das, 2019).

Efficacious nutrient management is beneficial in alleviating the growth-inhibiting effects of various biotic and abiotic agents including hypoxia. The scientific community is focusing on alternative approaches for the efficient and effective use of nutrients for sustainable crop production in a changing climate scenario. In this context, nanoparticles (NPs) are gaining popularity in the agriculture sector throughout the world. NPs are submicroscopic particles of 1–100 nm in size and are chemically synthesized or biosynthesized from various substances (Kah et al., 2018). They have multiple applications as nanofertilizers, non-herbicides, nanopesticides, and nanobiosensors. NPs of various substances such as Ag, Fe, Cu, Si, Al, Zn, ZnO, MnO, MgO, NiO, TiO_2_, CeO_2_, and Al_2_O_3_ have been shown to have beneficial uses in various fields such as environmental science, pharmacy, energy, and agriculture. NPs have greater efficiency than regular particles because of their higher surface area-to-volume ratio, multiple functions, high reactivity, high stability, and adsorption potential (Chhipa, 2019; Singh and Singh, 2019). The projected production of nanomaterials may reach 58,000 tons per annum by 2020 because of their extensive applications in agriculture as nanofertilizers, non-herbicides, nanopesticides, and nanobiosensors (Aslani et al., 2014).

In the past few decades, silicon (Si) has emerged as one of the beneficial elements that mitigate various abiotic stresses in plants such as nickel-induced oxidative stress (Abd_Allah et al., 2019), NaCl toxicity (Ahmad et al., 2019), cadmium stress (Jan et al., 2018; Kaya et al., 2020a), boron toxicity (Kaya et al., 2020b), fluoride stress (Banerjee et al., 2021), and drought stress (Bukhari et al., 2020). In addition, Si compensates phosphorus deficit-induced growth inhibition (Zhang et al., 2019) and alleviates combined salinity and cadmium stress in date palm (Khan et al., 2020). It is noteworthy to mention that all these abiotic stress regulations are achieved by modulating the eccentric physiological and molecular mechanism in different plants. The beneficial effects of Si on plant growth, development, and physiological regulation against certain abiotic and biotic stresses have been assessed; however, little is known about the role of SiNPs in the regulation of abiotic stresses in plants (Tripathi et al., 2017). Because of their unique properties, SiNPs exhibit great potential in agriculture and may help to mitigate the adverse effects of different abiotic stresses in plants (Abdel-Haliem et al., 2017). SiNPs have distinctive physiological characteristics that allow them to enter plant tissues and influence their metabolic activities. SiNPs may interact directly with plants and affect their morphology and physiology in various ways and improve plant growth and yield (Rastogi et al., 2019).

Globally, grapes are considered one of the most economically important and vital fruit species and grown in almost 100 countries. Grapes are consumed in different forms, as fresh, dried (raisins), and processed food and beverages (juices and soft drinks) (Signorelli et al., 2019). The current climatic anomalies and scenarios are a threat and can lead to unpredicted and urgent provocations to the grapevine’s traditional cultivation (Leolini et al., 2018). Muscadine grape (*Muscadinia rotundifolia* Michx.) is native to the southeastern United States, locally grown in North America. It is gaining popularity in southeastern states such as Florida, Alabama, Georgia, Mississippi, and Texas because of its sustainable production. One of the challenges to muscadine grapes production is their poor growth in calcareous soils or soils with low drainage capacity. Florida growers face flooding issues because of hurricanes and heavy rains every year. Crops under prolonged flooding suffer hypoxia or low oxygen conditions, and this problem becomes more severe in soils with low drainage.

Limited literature is available on the physiological response of muscadine grapes to flooding. Moreover, no literature is available on the effects of SiNPs on muscadine physiology under hypoxia stress. Thus, the study demonstrated here aimed to investigate the physiological and biochemical alterations in muscadine grapes exposed to hypoxia stress and to understand the physiological and biochemical mechanism associated with SiNPs-induced hypoxia tolerance in muscadine. We also appraised the hypothesis that SiNPs are more effective than conventional Si in alleviating the oxidative and osmotic stress in hypoxia-stressed muscadine plants.

## Materials and Methods

### Plant Material and Treatments

The experiment was carried out in the Environmental Horticulture Department’s greenhouses at the University of Florida, Gainesville, FL, United States. Five-month-old muscadine plants (cv. Alachua) were obtained from AgriStarts, Orlando, FL, United States, and then shifted to the hydroponic system. Each hydroponic unit comprised a 20-L bucket having two plants. All plants were grown in half-strength Hoagland solution for 1 week to overcome any stress during transplantation. The nutrient solution comprised macronutrients: 0.2 M Ca(NO_3_)_2_, 0.09 M MgSO_4_, 0.4 M KH_2_PO_4_, 0.01 M FeSO_4_, and 0.3 M KNO_3_, and micronutrients: 0.4 mM CuSO_4_, 1.4 mM ZnSO_4_, 0.5 mM H_3_BO_3_, and 10 mM H_2_MoO_4_. During the greenhouse experiment, the average daytime temperature was 24°C, the night temperature was 20°C, and the relative humidity was 85–90%. The photoperiod was maintained at 12 h by using cool-white fluorescent lamps with a photon flux density of 490 μmol m^–2^ s^–1^.

Afterward, six treatments were applied, i.e., (i) control (aerated plants), (ii) aerated + Si (250 ppm), (iii) aerated + SiNP (250 ppm), (iv) hypoxia stress, (v) hypoxia stress + Si (250 ppm), and (vi) hypoxia stress + SiNPs (250 ppm). In a preliminary study (data not shown), the level of conventional Si (250 ppm) was found to be very effective in improving muscadine plant growth, so to maintain the uniformity, an equal concentration of SiNPs (250 ppm) was used. Each treatment was replicated four times, and each replicate comprised 10 plants (five containers per replicate in each treatment). Hypoxia stress was induced by limiting the oxygen supply to the nutrient solution in hydroponic units. The aeration was carried out in the control treatment using the small tubing attached with air pumps (Deluxe LGPUMPAIR38). All treatments were randomly organized in each replication. The average concentration of dissolved oxygen in aerated and hypoxia-stressed (non-aerated) hydroponic units was maintained at 8.8 and 1.75 mg/L, respectively, and monitored daily using an oxygen meter (HI98193, Hanna Instruments, Smithfield, RI, United States).

Conventional Si (SiO_2_) and SiNP of 20–30 nm size were obtained from Sigma–Aldrich and US Research Nanomaterials Inc., Houston, TX, United States, respectively. Si from both sources (conventional Si and SiNPs) was continuously supplemented to the respective plants after mixing with the nutrient solution. The pH of the hydroponic units’ solution was maintained at 4.5–5.0 and tested each day using a portable pH meter (HI9124, Hanna Instruments, Smithfield, RI, United States). Any fluctuation in pH was adjusted by using NaOH or HCl. The nutrient solution was replaced on a weekly basis throughout the experiment.

After 3 weeks of hypoxia stress, data on leaf greenness and gas exchange characteristics were determined. After photosynthesis and leaf greenness measurements, destructive sampling was performed for enzymatic, osmolyte, and nutrient attributes.

### Plant Biomass

At the end of the experiment (5 weeks after hypoxia stress), the plants (four plants per replicate) were gently removed from hydroponic units and washed with distilled water. The plants were blotted out with filter paper to remove any water present on the leaves, stems, and roots. The plant tissues were then oven-dried (Memmert-110, Schawabach, Germany) at 65°C for 4 days, and the average dry weight was recorded. After recording dry weights, the samples were ground for further chemical analysis.

### Leaf Gas Exchange Parameters and Chlorophylls

Photosynthetic activity, stomatal conductance, and internal CO_2_ levels were measured using LiCor (LI 6400 XT, Licor Corporation, Lincoln, NE, United States) from fully expanded healthy leaves. Three leaves from the top, middle, and lower portions of the plants were used, and measurements were taken from four plants per replicate. The following conditions were observed during the determination of gas exchange attributes: molar airflow per unit leaf area 387 mmol m^–2^ s^–1^, leaf temperature 23.5°C, air CO_2_ concentration 394 μmol mol^–1^, atmospheric pressure 89.7 kPa, and photosynthetic photon flux density 1750 μmol m^–2^ s^–1^. All gas exchange parameters were recorded between 9:00 and 11:00 h. The leaf greenness was measured on the leaves’ adaxial surface using a SPAD meter (SPAD-501, Minolta, Inc., Kyoto, Japan). SPAD measurements were taken from five leaves at the top, middle, and lower end of the plant, and four plants per treatment were used. So, a total of 60 leaves per treatment were used for leaf greenness.

### Antioxidant Activities Determination

To determine the antioxidant activities of superoxide dismutase (SOD), peroxidase (POD), and catalase (CAT), 0.5 g of leaf and root tissue was ground in an ice-cooled tissue grinder containing prechilled 5 mL of 50 mM phosphate buffer (pH 7.8). The resulting homogeneous mixture was centrifuged at 15,000 × *g* for 20 min at 4°C, and the supernatant was used to determine the antioxidant activities. The SOD activity was measured by assessing the potential to hinder the photoreduction of nitroblue tetrazolium as described by Giannopolitis and Ries (1977). The CAT and POD activities were measured according to Maehly and Chance (1954) with some modifications. The CAT reaction solution (3 mL) contained 50 mM phosphate buffer (pH 7.0), 5.9 mM H_2_O_2_, and 0.1 mL of enzyme extract. Changes in the reaction solution’s absorbance at 240 nm were recorded every 20 s. One unit of CAT activity was defined as an absorbance change of 0.01 U min^–1^. The POD reaction solution (3 mL) contained 50 mM phosphate buffer (pH 5.0), 20 mM guaiacol, 40 mM H_2_O_2_, and 0.1 mL of enzyme extract. Changes in the absorbance of the reaction solution at 470 nm were recorded at every 20 s. One POD activity unit was defined as an absorbance change of 0.01 units per min. The activity of each enzyme was expressed based on the protein content.

Concentrations of ascorbic acid (AsA) and glutathione reductase (GSH) were determined after grinding 0.5 g ice-cooled plant tissue in liquid nitrogen; then, 1 mL of ice-cooled 2.5 M HClO_4_ was added to it. The crude extracts were centrifuged at 16,000 × *g* for 20 min at 4°C, and the supernatant was collected, aliquoted (400 μL each), and stored at −70°C until used. The AsA content was assayed by following the absorbance change at 265 after the addition of ascorbate oxidase (Arakawa et al., 1981). The GSH content was assayed following the change in absorbance at 412 after the addition of DTNB [5,5′-dithiobis (2-nitrobenzoic acid)] according to the method of Griffith (1980).

### Lipid Peroxidation, Reactive Oxygen Species, and Osmolytes

The Elstner’s procedure was used to determine lipid peroxidation, reactive oxygen species (ROS), and osmolyte levels. A homogenate of leaves or roots supplemented with 0.5 mL of phosphate buffer, 1 mL of xanthine oxidase, and 0.1 mL of hydroxyl ammonium chloride was incubated at 25°C for 20 min. A 0.5-mL aliquot of this mixture was then mixed with 0.5 mL of sulfanilic acid and 0.5 mL of α-nepthylamine and shaken vigorously. The mixture was kept at room temperature for 20 min, and the optical density was recorded at 530 nm with a spectrophotometer (Thermo Fisher Scientific, Waltham, MA, United States). The superoxide concentration was measured using a standard curve. Hydrogen peroxide (H_2_O_2_) was measured following the protocol of Patterson et al. (1984). Plant tissues were homogenized in acetone (1 g of tissue with 2 mL of acetone). Titanium reagent was added to the supernatant, and 17 M ammonia solution was added to the mixture. The precipitate was separated, washed with acetone, and dissolved in 3 mL of H_2_SO_4_ (2 N). The absorbance of the solution was determined at 410 nm and adjusted by subtracting the absorbance of a “blank” prepared by the same procedure but without plant tissue. Lipid peroxidation was estimated by measuring the concentrations of malondialdehyde (MDA) and thiobarbituric acid (TBA) as described by Heath and Packer (1968). Equal volumes of tissue extract and 0.5% (wt/vol) TBA solution containing 20% (wt/vol) trichloroacetic acid were mixed. The mixture was kept at 95°C for 30 min and then snap cooled in a cooling bath filled with ice. The mixture was then centrifuged at 3000 × *g* for 10 min. The absorbance of the supernatant at 532 and 600 nm was measured. The MDA concentration was calculated from its molar extinction coefficient (155 mM^–1^ cm^–1^) and expressed in μmol MDA mL^–1^ g^–1^ DW. MDA (nmol) = Δ (A 532 nm - A 600 nm)/1.56 × 105. The free proline content in leaves and roots was estimated by the method of Bates et al. (1973) and glycinebetaine (GB) by the method described by Grieve and Grattan (1983).

### Mineral Nutrient Concentration

Leaf and root samples were dried in a drying oven (Memmert-110, Schawabach, Germany) at 65°C for 4 days to determine the concentration of various nutrients. The concentrations (mmol g^–1^) of nitrogen (N), phosphorus (P), potassium (K), iron (Fe), and manganese (Mn) were determined using 0.5 g of root and leaf tissue ground with a mortar and pestle. Nitrogen was determined with a Perkin-Elmer PE 2400 CHN analyzer (Perkin-Elmer, Waltham, MA, United States). Samples for the determination of P, K, Fe, and Mn were ashed in a Muffle furnace (Thermo Fisher Scientific, Waltham, MA, United States) at 500°C for 4 h and then dissolved in 1 M HCl and analyzed, for P by an ammonium molybdate–AsA procedure (Murphy and Riley, 1962), for K by atomic emission spectrometry and Zn, Fe, and Mn by atomic absorption spectrometry.

### Statistical Analysis

A one-way analysis of variance and Fisher least significance difference (LSD) *post hoc* test were used to determine the level of significant differences between the treatments. All data were analyzed using STATISTICA 9.0 (Stat-Soft, Inc., United States). There were four replications per treatment, and each replicate comprised eight plants (two plants per hydroponic system).

## Results

### Effects of Si and SiNP on Plant Dry Biomass

**FIGURE 1:**
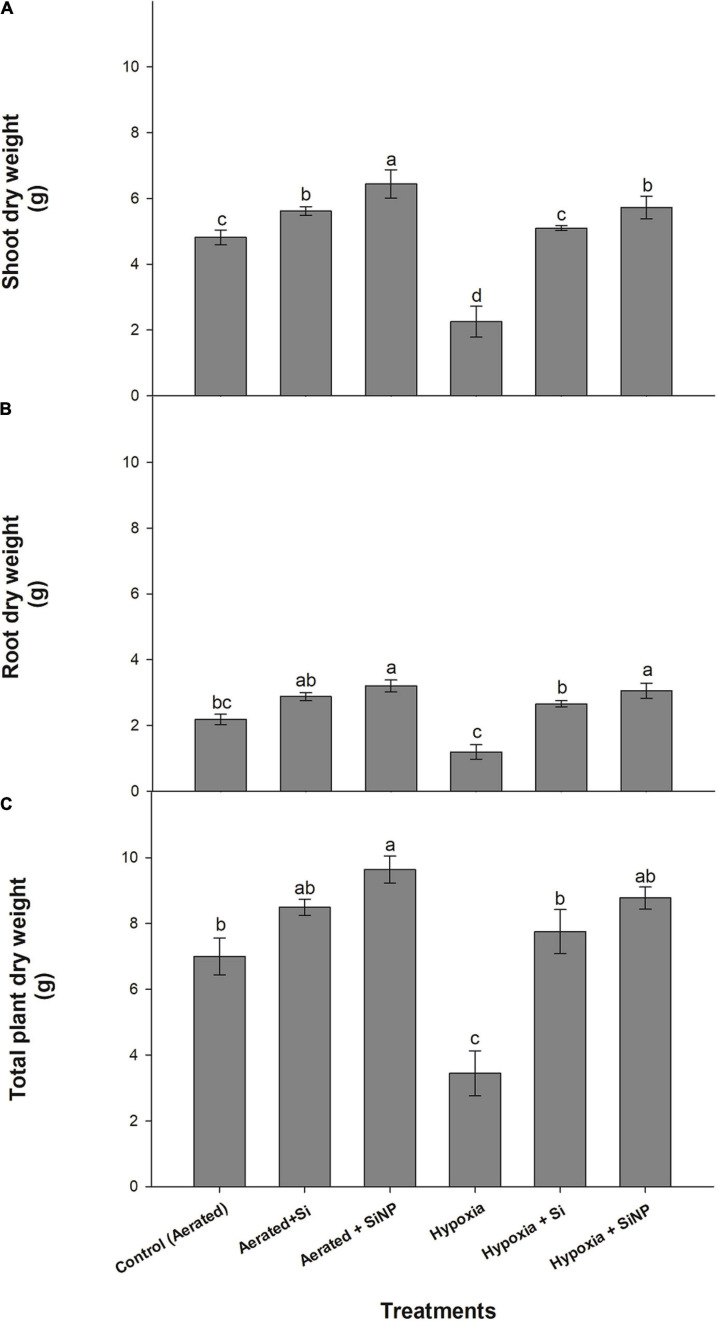
Shoot dry **(A)**, root dry **(B)**, and total plant dry **(C)** biomass (g per plant) of muscadine grape plants grown hydroponically under controlled greenhouse conditions with six treatments, i.e., control (aerated), aerated + Si (250 ppm), aerated + SiNPs (250 ppm), hypoxia (no aeration in nutrient solution), hypoxia + Si (250 ppm), and hypoxia + SiNPs (250 ppm). Values are mean of four independent replicates ± SE (*n* = 4). Values with different letters differ significantly for *p* < 0.05, using the least significant difference (LSD). The vertical bar indicates the standard error of the mean (*n* = 4). Si, conventional silicon; SiNPs, silicon nanoparticles of size 20–30 nm.

The induced hypoxia stress led to a severe reduction of 50% in shoot dry weight and 45% in the root dry weight, whereas a decrease of 50% in the total dry weight concerning the control (aerated) plants was observed. Conventional Si improved the growth attributes in control plants by improving root dry weight (15%), shoot dry weight (30%), and total dry weight (20%). Whereas plants grown under hypoxia stress but supplemented with conventional Si had an increase in their root dry weight, shoot dry weight, and total dry weight by 125, 120, and 125%, respectively, compared to those grown under hypoxia stress without Si. However, SiNPs yielded a better improvement in control plants (by improving root dry weight by 30%, shoot dry weight by 46%, and total dry weight by 20%) and hypoxia-stressed plants (with an overall improvement of all the parameters by approximately 155%). Overall, SiNPs proved to be the most effective in improving plant biomass under aerated conditions and hypoxia stress (Figure 1).

### Effects of Si and SiNP on Leaf Greenness and Gas Exchange Attributes

**FIGURE 2:**
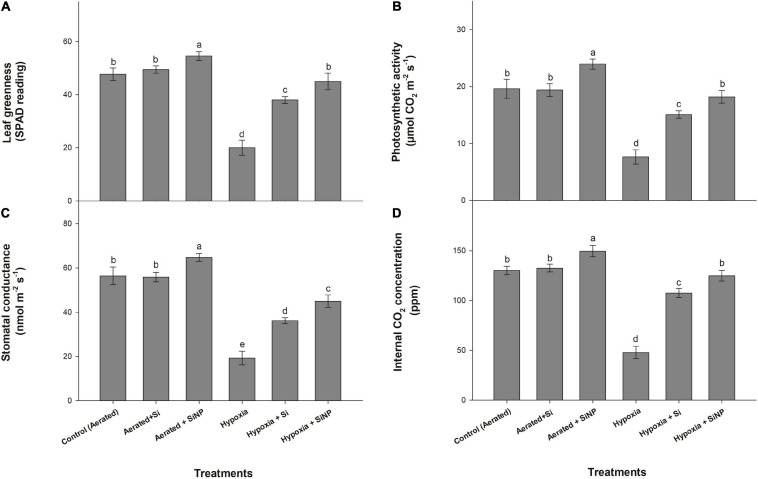
Leaf greenness **(A)**, photosynthetic activity **(B)**, stomatal conductance **(C)**, and internal CO_2_ concentration **(D)** of muscadine grape plants grown hydroponically under controlled greenhouse conditions with six treatments, i.e., control (aerated), aerated + Si (250 ppm), aerated + SiNPs (250 ppm), hypoxia (no aeration in nutrient solution), hypoxia + Si (250 ppm), and hypoxia + SiNPs (250 ppm). Values are mean of four independent replicates ± SE (*n* = 4). Values with different letters differ significantly for *p* < 0.05, using the least significant difference (LSD). The vertical bar indicates the standard error of the mean (*n* = 4). Si, conventional silicon; SiNPs, silicon nanoparticles of size 20–30 nm.

Hypoxia stress caused a 60% reduction in leaf greenness in comparison to the control plants. However, Si- and SiNP-treated plants were able to overcome this stress. SiNPs proved to be more effective with a 120% improvement in leaf greenness than Si, with an increase in SPAD reading by 90% (Figure 2). Both Si and SiNPs did not cause any significant effect on leaf greenness in control (aerated) plants.

Photosynthetic activity in leaves decreased by 60% under hypoxia stress. However, the negative effect of hypoxia stress on photosynthesis was mitigated by applying both forms of Si. Plants treated with conventional Si had a 97% improvement in photosynthetic activity under hypoxia stress, but control (aerated) plants did not show any significant effect of Si on photosynthesis (Figure 2), whereas SiNP-treated plants had a 20 and 145% increase in photosynthetic activity in control and hypoxia conditions, respectively.

Hypoxia caused a reduction of 65% in stomatal conductance. The plants treated with Si and SiNPs maintained higher stomatal conductance values as compared to non-treated plants. Conventional Si led to an 87% improvement in hypoxia-stressed plants’ stomatal conductance, whereas no significant differences were witnessed in control (aerated) plants. On the other hand, SiNPs exhibited more efficacy by enhancing stomatal conductance by 14% in control plants and 133% in hypoxia-stressed plants (Figure 2).

Plants grown under hypoxia stress also showed a decline (of 60%) in internal CO_2_ concentration. Exogenous application of Si and SiNPs counteracted the negative effect of hypoxia on internal CO_2_ concentration. Si improved CO_2_ concentration by 15% in aerated and 125% in hypoxia-stressed plants, while SiNPs improved internal CO_2_ level by 10 and 160% in control (aerated) and hypoxia-stressed plants, respectively.

### Effects of Si and SiNP on Activities of Antioxidant

**FIGURE 3:**
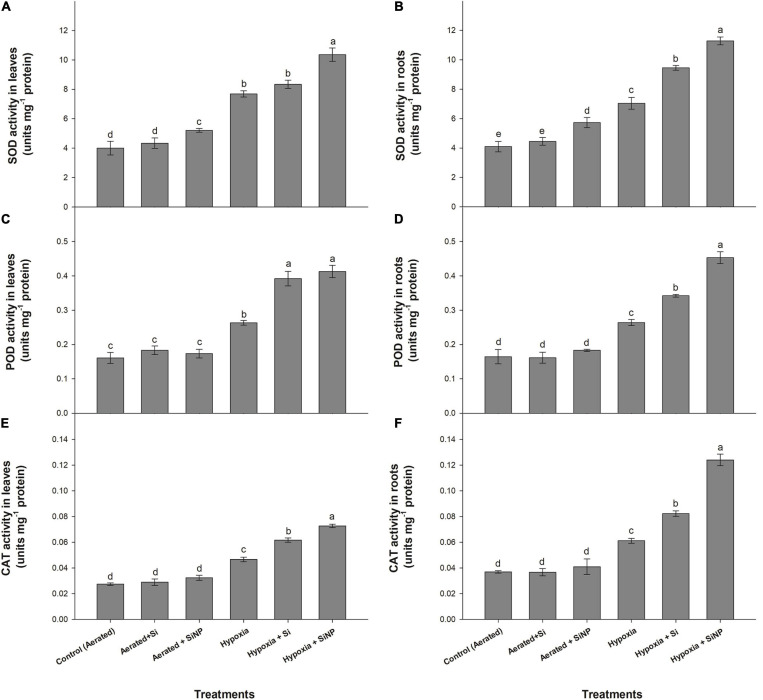
SOD **(A,B)**, POD **(C,D)**, and CAT **(E,F)** activities quantified in leaves and roots of muscadine grape plants grown hydroponically under controlled greenhouse conditions with six treatments, i.e., control (aerated), aerated + Si (250 ppm), aerated + SiNPs (250 ppm), hypoxia (no aeration in nutrient solution), hypoxia + Si (250 ppm), and hypoxia + SiNPs (250 ppm). Values are mean of four independent replicates ± SE (*n* = 4). Values with different letters differ significantly for *p* < 0.05, using the least significant difference (LSD). The vertical bar indicates the standard error of the mean (*n* = 4). Si, conventional silicon; SiNPs, silicon nanoparticles of size 20–30 nm; SOD, superoxide dismutase; POD, peroxidase; CAT, catalase.

The induced hypoxia stress led to an increase in the tested antioxidants’ enzymatic activity, both in leaves and roots. Leaf and root SOD activities were found to increase by 90 and 70%, POD activities by 65 and 60%, and CAT activities by 70 and 65%, respectively, under hypoxia stress (Figure 3).

Si application was not very effective under aerated conditions (no hypoxia), resulting in about 8% increase in SOD content of both leaves and roots and improved POD activity in leaves by 13%, whereas it had no significant effect on POD activity in roots and CAT contents (both of leaves and roots). Under hypoxia conditions, conventional Si improved SOD activity by 8% in leaves, whereas the SOD of roots improved by 30%. POD activity was increased by 50 and 30% in leaves and roots, respectively. CAT activity was increased by 30 and 35% in leaves and roots, respectively, in response to Si application under hypoxia stress.

Si nanoparticles were more efficacious than conventional Si in augmenting SOD, POD, and CAT activities in leaf and root tissues. Similar to Si, SiNPs also accelerated antioxidants activities more in hypoxia-stressed plants than those under aerated conditions. Leaves and roots of plants treated with SiNPs had an increase in SOD activities by 60 and 55%, POD activities by 70 and 55%, and CAT activities by 55 and 100% under hypoxia conditions (Figure 3).

### Effects of Si and SiNP on Non-enzymatic Antioxidants

Non-enzymatic antioxidants (AsA and GSH) were also increased in roots and leaves under hypoxia stress. AsA contents were increased by 35 and 90%, whereas GSH contents increased by 25 and 85% in leaves and roots, respectively, in response to hypoxia stress.

**FIGURE 4:**
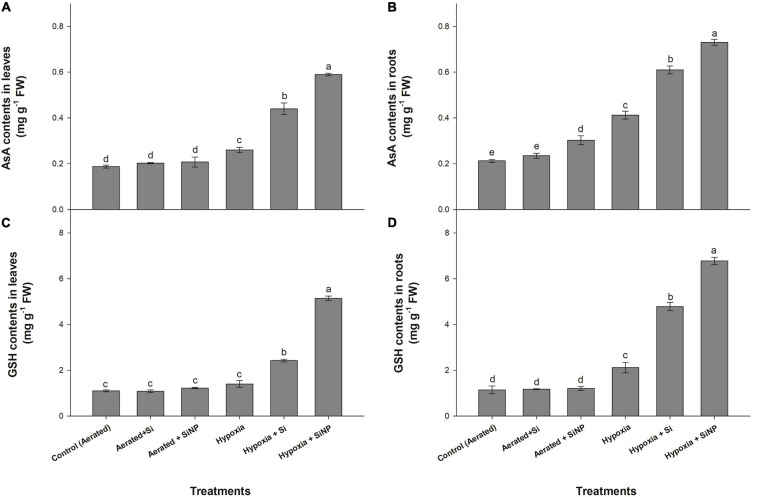
AsA **(A,B)** and GSH **(C,D)** contents quantified in leaves and roots of muscadine grape plants grown hydroponically under controlled greenhouse conditions with six treatments, i.e., control (aerated), aerated + Si (250 ppm), aerated + SiNPs (250 ppm), hypoxia (no aeration in nutrient solution), hypoxia + Si (250 ppm), and hypoxia + SiNPs (250 ppm). Values are mean of four independent replicates ± SE (*n* = 4). Values with different letters differ significantly for *p* < 0.05, using the least significant difference (LSD). The vertical bar indicates the standard error of the mean (*n* = 4). Si, conventional silicon; SiNPs, silicon nanoparticles of size 20–30 nm; AsA, ascorbic acid; GSH, glutathione reductase.

Under aerated conditions, both forms of Si (Si and SiNPs) did not show any significant effect on AsA and GSH contents (Figure 4). Contrary to aerated conditions, both Si and SiNPs substantially increased the levels of AsA and GSH under hypoxia stress. In Si-treated plants, AsA contents increased by 70 and 45%, whereas GSH contents increased by 70 and 125% in leaves and roots, respectively. SiNP-treated plants substantially enhanced the levels of AsA with 120 and 75%, whereas 265 and 220% of GSH were enhanced in leaves and roots, respectively (Figure 4).

### Effects of Si and SiNP on Proline and GB Accumulation

**FIGURE 5:**
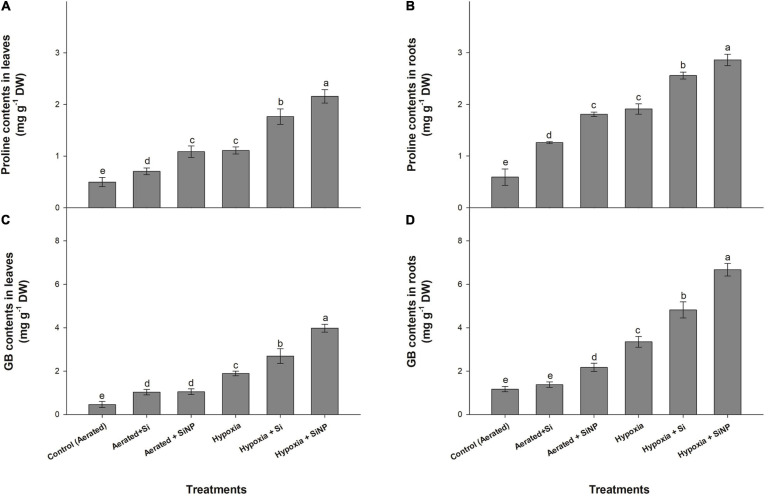
Proline **(A,B)** and GB **(C,D)** contents quantified in leaves and roots of muscadine grape plants grown hydroponically under controlled greenhouse conditions with six treatments, i.e., control (aerated), aerated + Si (250 ppm), aerated + SiNPs (250 ppm), hypoxia (no aeration in nutrient solution), hypoxia + Si (250 ppm), and hypoxia + SiNPs (250 ppm). Values are mean of four independent replicates ± SE (*n* = 4). Values with different letters differ significantly for *p* < 0.05, using the least significant difference (LSD). The vertical bar indicates the standard error of the mean (*n* = 4). Si, conventional silicon; SiNPs, silicon nanoparticles of size 20–30 nm; GB, glycinebetaine.

Proline and GB contents increased in plants in response to hypoxia stress. Hypoxia-stressed plants exhibited an increase in proline accumulation in leaves and roots by 120% and 220%, whereas GB accumulation was increased by 300 and 180% in leaves and roots, respectively. Control (aerated) plants treated with Si had improved proline content by 40 and 110% in leaves and roots, whereas GB accumulation was increased by 120 and 15%, respectively. However, plants grown under hypoxia stress and supplemented with conventional Si had an increase in proline (60% in leaves and 30% in roots) and GB (40% in leaves and roots) compared to hypoxia-stressed plants without Si (Figure 5).

Si nanoparticles were found to be more effective compared to simple Si in improving proline (115% in leaves and 200% in roots) and GB accumulation (125% in leaves and 85% in roots) in plants grown under controlled conditions. Hypoxia-stressed plants treated with SiNPs had a higher increase in proline (90 and 50% in leaves and roots, respectively) and GB contents (110 and 100% in leaves and roots, respectively) than those grown under hypoxia stress but no SiNP (Figure 5).

### Effects of Si and SiNP on Lipid Peroxidation and Generation of Reactive Oxygen

**FIGURE 6:**
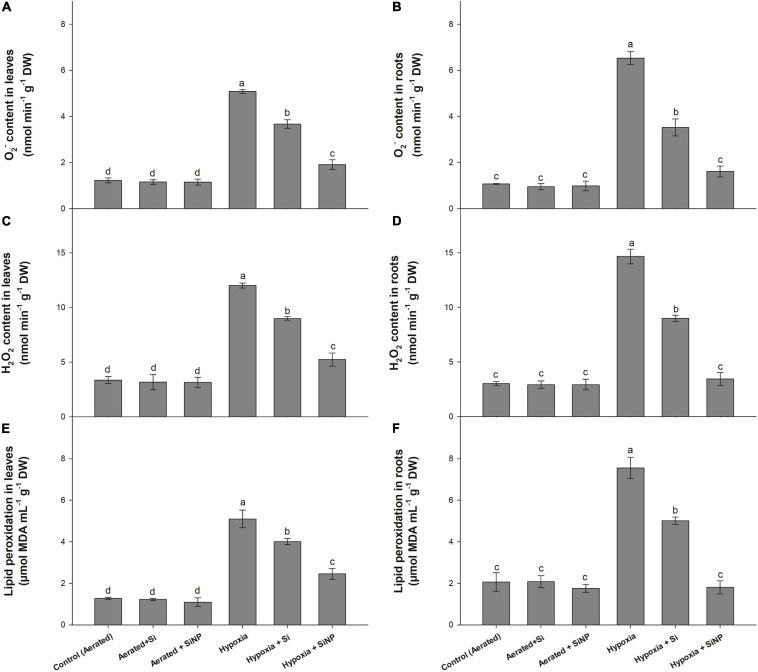
O_2_^–^ content **(A,B)**, H_2_O_2_ content **(C,D)** and rate of lipid peroxidation **(E,F)** quantified in leaves and roots of muscadine grape plants grown hydroponically under controlled greenhouse conditions with six treatments, i.e., control (aerated), aerated + Si (250 ppm), aerated + SiNPs (250 ppm), hypoxia (no aeration in nutrient solution), hypoxia + Si (250 ppm), and hypoxia + SiNPs (250 ppm). Values are mean of four independent replicates ± SE (*n* = 4). Values with different letters differ significantly for *p* < 0.05, using the least significant difference (LSD). The vertical bar indicates the standard error of the mean (*n* = 4). Si, conventional silicon; SiNPs, silicon nanoparticles of size 20–30 nm; O_2_^–^, superoxide radical; H_2_O_2_, hydrogen peroxide.

Hypoxia stress led to the generation of ROS in the leaf and root tissues of muscadine grape plants. Higher concentrations of O_2_^–^ and H_2_O_2_ were recorded in hypoxia-stressed plants compared to aerated plants. Plants under hypoxia stress had higher ROS levels, so a high rate of lipid peroxidation in the leaves and root tissues was observed. However, Si supplementation suppressed the formation of ROS and, consequently, lowered the lipid peroxidation. Conventional Si suppressed the production of O_2_^–^ (by 25 and 45%, in roots and leaves) and H_2_O_2_ (by 25 and 35% in roots and leaves) and the rate of lipid peroxidation (by 20 and 30%, in root and leaves). Again SiNPs yielded better performance by reducing the formation of O_2_^–^ (by 60 and 75% in roots and leaves) and H_2_O_2_ (by 55 and 75% in roots and leaves) and rate of lipid peroxidation (50 and 75% in root and leaves) (Figure 6) as compared to plants grown without SiNP under hypoxia stress.

### Effects of Si and SiNP on the Concentration of Various Nutrients

**FIGURE 7:**
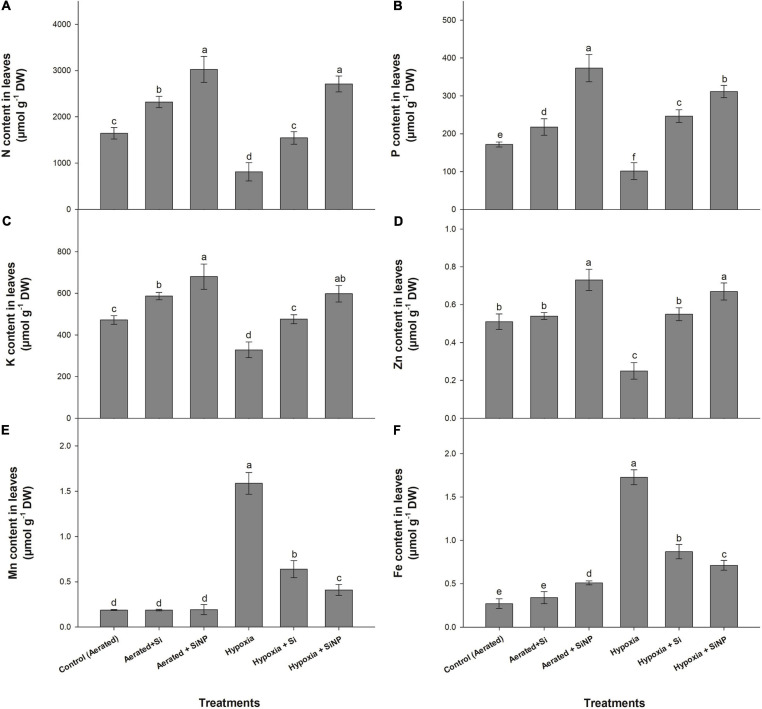
N **(A)**, P **(B)**, K **(C)**, Zn **(D)**, Mn **(E)**, and Fe **(F)** contents quantified in leaves of muscadine grape plants grown hydroponically under controlled greenhouse conditions with six treatments, i.e., control (aerated), aerated + Si (250 ppm), aerated + SiNPs (250 ppm), hypoxia (no aeration in nutrient solution), hypoxia + Si (250 ppm), and hypoxia + SiNPs (250 ppm). Values are mean of four independent replicates ± SE (*n* = 4). Values with different letters differ significantly for *p* < 0.05, using the least significant difference (LSD). The vertical bar indicates the standard error of the mean (*n* = 4). Si, conventional silicon; SiNPs, silicon nanoparticles of size 20–30 nm.

Plants under hypoxia stress had a reduction in N (50%), P (40%), K (30%), and Zn (50%) in their leaves, but the level of Mn and Fe was drastically increased in leaves compared to control plants. Although hypoxia stress limited nutrient uptake, both Si and SiNPs have had a positive effect on improving nutrient uptake, so the plants applied with SiNPs maintained higher levels of N, P, and K (Figure 7).

Plants grown under aerated conditions (control) and treated with Si improved N, P, and K levels by 40, 26, and 24%, respectively. Under hypoxia stress, plants with Si application showed a 90, 140, 45, and 120% increase in N, P, K, and Zn. The unusual increase in the levels of Mn and Fe was observed under hypoxia stress, but this increase was decreased in Si-treated plants (Figure 7).

Likewise, SiNPs improved the concentration of N (80%), P (115%), K (45%), and Zn (35%) in aerated plants, whereas under hypoxia conditions, SiNPs increased N, P, K, and Zn by 230, 200, 80, and 165%, respectively, compared to plants grown without SiNP application under hypoxia stress (Figure 7).

### Effects of Si and SiNP on the Concentration of Si

**FIGURE 8:**
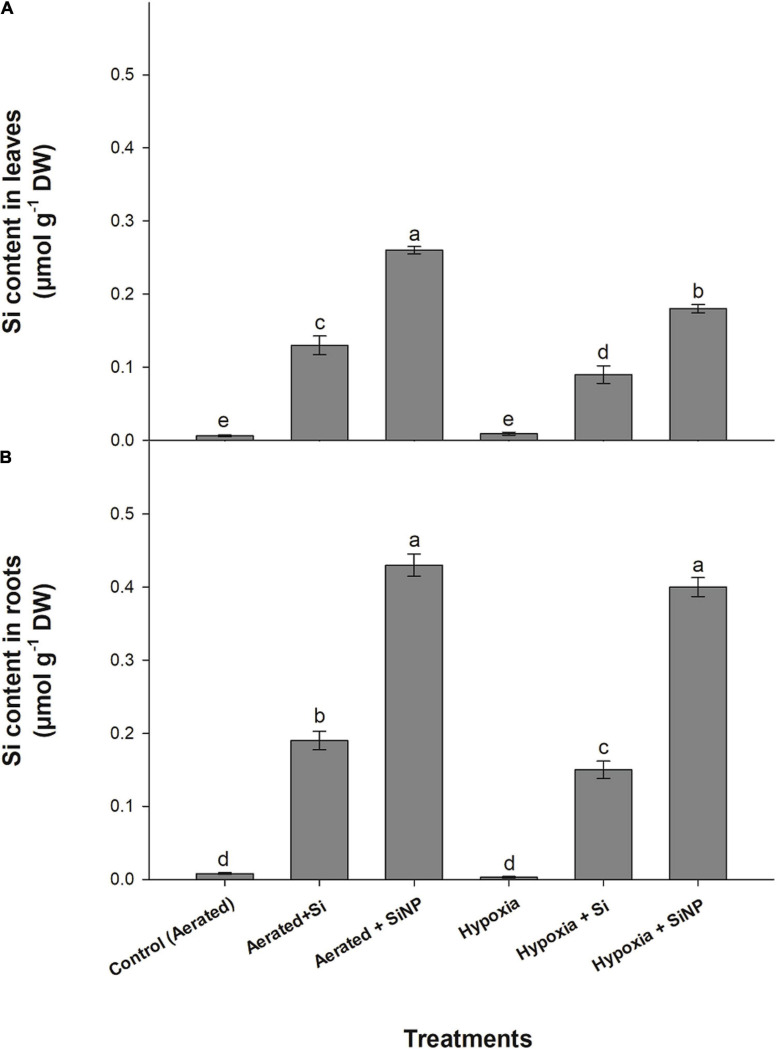
Si contents quantified in leaves **(A)** and roots **(B)** of muscadine grape plants grown hydroponically under controlled greenhouse conditions with six treatments, i.e., control (aerated), aerated + Si (250 ppm), aerated + SiNPs (250 ppm), hypoxia (no aeration in nutrient solution), and hypoxia + Si (250 ppm), and hypoxia + SiNPs (250 ppm). Values are mean of four independent replicates ± SE (*n* = 4). Values with different letters differ significantly for *p* < 0.05, using the least significant difference (LSD). The vertical bar indicates the standard error of the mean (*n* = 4). Si, conventional silicon; SiNPs, silicon nanoparticles of size 20–30 nm; O_2_^–^, superoxide radical; H_2_O_2_, hydrogen peroxide.

Exogenous application of Si and SiNP enhanced the level of Si in leaf tissues of muscadine plants grown under hypoxia stress. Plants treated with SiNPs had a higher concentration of Si in their leaves than those treated with Si. The supplementation of Si increased the Si contents inside the leaves and roots by five times more than aerated plants and increased by three times more in hypoxia-stressed plants. On the other hand, plants applied with SiNPs showed an increase in Si content by 100 and 125% under aerated conditions and showed 10 times’ increase in hypoxia-stressed plants (Figure 8).

## Discussion

The exogenous application of Si ameliorates various abiotic stresses by modulating the physiological and biochemical characteristics of a wide array of crop plants (Jang et al., 2018). Si has been proven very effective in mitigating hypoxia-induced deleterious effects in crops (Debona et al., 2017). Nanofertilizers are becoming increasingly popular in tackling various biotic and abiotic stresses and the complications associated with them in crops. The high efficacy of SiNPs in ameliorating abiotic stress compared to conventional Si in cereal crops has been demonstrated in plants (Tripathi et al., 2017; Namjoyan et al., 2020).

Severe reductions in the biomass of muscadine grape plants were observed under hypoxia stress. Our findings coincide with the earlier report, in which waterlogging (hypoxia) adversely affected the development of root biomass and subsequently led to compromised plant growth and development (de San Celedonio et al., 2016). Under hypoxia conditions, dispersal of oxygen is 320K times lower than in aerated soils, which ultimately hinders plant growth (Barickman et al., 2019; da Ponte et al., 2019). However, our findings revealed that the use of Si and SiNPs could improve the growth and dry biomass of muscadine grape plants. Si has been found to be a growth-promoting element in grapevine (Zhang et al., 2017). SiNPs also improve growth, development, and associated characters such as fresh weight, dry weight, and shoot length in peppermint (Ahmad et al., 2020), banana (Mahmoud et al., 2020), and other plants, assumingly by boosting the production of organic compounds such as proteins, chlorophyll contents, polyamines, and phenolic compounds. In addition, SiNPs improve plant growth and yield by morphological, physiological, and biochemical modulations (Rastogi et al., 2019) either directly or indirectly affecting plant growth and development-related parameters (Siddiqui and Al-Whaibi, 2014). This study demonstrated that SiNP-treated muscadine plants maintained growth and development by modulating higher green pigments, stomatal conductance, internal CO_2_, and photosynthetic activity, thereby enabling them to produce biomass, particularly under stressed conditions.

Oxygen deficiency limits plants’ gas exchange attributes and results in an impaired photosynthetic activity (He et al., 2018; Barickman et al., 2019). In this study, hypoxia stress caused a reduction in green pigments, stomatal conductance, and internal CO_2_ concentration. Changes in gas exchange parameters represent a highly integrative basis for hypoxia’s overall effect on photosynthetic activity. Reduced photosynthetic activities could have been due to hypoxia-induced responses related to ethylene production, possibly causing stomatal closure (Bashar et al., 2019). Low oxygen conditions also alter oxidation–reduction reactions in the chloroplast’s cellular and subcellular compartments, resulting in reduced gas exchange and photosynthetic activity. On the other hand, the chloroplast is a crucial photosynthetic organelle and one of the potential sites for ROS production under stressed conditions. Excessive amounts of ROS cause severe damage to thylakoid membranes and degradation of chlorophyll molecules, leading to reduced light absorption and ATP production (Sairam and Srivastava, 2002; Wang et al., 2018; Anee et al., 2019). The exogenous application of SiNPs is very effective in improving the gas exchange characteristics of hypoxia-stressed muscadine plants. SiNPs-treated plants had reduced oxidative damage in leaf tissues, indicating that SiNPs safeguarded their photosynthetic apparatus and maintained high photosynthetic activity under hypoxia stress. SiNPs-treated plants showed more hypoxia tolerance and high photosynthetic activity and gas exchange than Si-treated plants. High hypoxia tolerance of SiNP-treated plants may have been attributed to high enzymatic and non-enzymatic antioxidant activities resulting in reduced oxidative damage to cells. Various reports have indicated that SiNPs improved chlorophyll content and photosynthetic efficiency both under normal and stressed conditions (Cao et al., 2015; Ahmad et al., 2020; Mahmoud et al., 2020).

Organic osmolytes, such as proline and GB, play an important role in osmoregulation and stress mitigation under various plants’ stressed conditions (Funck et al., 2012). A strong correlation between osmoprotectants’ concentration and stress tolerance has been observed and is considered one of the potential stress tolerance indicators (Sharma et al., 2019). Proline and GB help to alleviate stress-induced adverse effects by regulating the formation of important proteins such as Rubisco, safeguarding the photosynthetic apparatus, maintaining the redox balance, improving membrane stability, and scavenging the ROS (Kavi Kishor and Sreenivasulu, 2014; Zhang and Becker, 2015; Annunziata et al., 2019). Muscadine plants exposed to hypoxia stress showed an increase in the accumulation of proline and GB in leaf and root tissues compared to the control (aerated) plants. Increased proline and GB concentrations in leaves and roots of muscadine plants under hypoxia stress are an adaptation mechanism to withstand oxygen deficiency in flooding conditions. A further augmentation in the accumulation of proline and GB was observed upon treating the plants with Si and SiNPs. Hypoxia stress causes osmotic stress and reduces root permeability to water, ultimately leading to a decline in root hydraulic conductance and stomal closure (Clarkson et al., 2000). This could also be the other reason for reduced stomatal conductance in hypoxia-stressed muscadine plants. Under stressed conditions, osmoprotectants such as proline and GB aid cells in osmotic adaptation and establish an osmotic gradient to regulate plants’ water uptake capacity (Ashraf and Foolad, 2007). In this study, higher accumulations of proline and GB in response to both forms of Si (conventional Si and SiNPs) might have contributed to mediate plant water status and hydraulic conductivity during hypoxia stress. Hypoxia-stressed plants supplemented with SiNPs exhibited higher proline and GB accumulation in their leaves and roots than those grown with conventional Si. SiNPs have already been reported to improve osmolytes’ accumulation under certain abiotic stress conditions (Fatemi et al., 2020; Banerjee et al., 2021). As proline also acts as a strong non-enzymatic antioxidant (Das and Roychoudhury, 2014), and SiNP-treated plants had a high accumulation of proline, it could have contributed to ROS scavenging to alleviate oxidative damage in the leaf and root tissues.

Reactive oxygen species are produced in plants as a byproduct of cellular metabolism, but abiotic stresses (e.g., salinity, drought, heat, and flooding) lead to the overproduction of ROS that causes oxidative stress. Oxidative stress is assessed by determining the rate of generation of ROS (O_2_^–^, H_2_O_2_, O, α-O, and OH^–^) and lipid peroxidation (Mittler, 2002). Although ROS serve as signaling molecules, but they induce oxidative cell death if an imbalance between ROS formation and their elimination occurred (Kapoor et al., 2019). Plants have a defensive system comprising various enzymatic antioxidants (such as SOD, POD, CAT, APX, and GPX) and non-enzymatic antioxidants (like AsA, proline, phenolic compounds, carotenoids, flavonoids, and glutathione) to alleviate ROS-induced oxidative damage (Kapoor et al., 2019). The antioxidative system scavenges ROS by converting its toxic forms into non-toxic ones. In the current investigation, hypoxia stress induced oxidative damage in muscadine plants by increasing O_2_^–^ and H_2_O_2_ levels, resulting in a high rate of lipid peroxidation in leaves and roots. A positive correlation between the level of ROS and the rate of lipid peroxidation was observed in muscadine plants exposed to hypoxia stress, thereby exhibiting strong relationship between ROS levels and lipid peroxidation. A high rate of lipid peroxidation is suggestive of increased cell death and chlorophyll degradation, which consequently reduces photosynthetic activity in leaves of hypoxia-stressed plants (Foyer, 2018). Muscadine plants counteracted hypoxia stress by activating the antioxidative defensive system, so plants under hypoxia stress showed slightly increased activities of enzymatic antioxidants (SOD, POD, and CAT) and concentration of non-enzymatic antioxidants (AsA, GSH, and proline) in leaves and roots. Exogenous application of SiNPs further strengthened the ROS scavenging system by accelerating enzymatic and non-enzymatic antioxidant activities in stressed plants, thus mitigating hypoxia-induced oxidative damage in leaves and roots. Studies have shown that SiNP mitigated oxidative damage by regulating the activities of both enzymatic and non-enzymatic antioxidants in various plant species under different abiotic stresses (Sattar et al., 2019; Namjoyan et al., 2020), and our results are consistent with these reports. High antioxidant activities and levels of the non-enzymatic antioxidant compound in SiNP-treated plants could have been associated with higher accumulation of Si in plant tissues, as SiNPs-treated plants had higher Si contents in leaves than conventional Si-treated plants.

Reduction in nutrient uptake and their translocation are the significant physiological effect of hypoxia in most plant species (Slowik et al., 1979). These nutrients deficiencies are the result of poor root hair development, less root elongation, alterations in root hydraulic conductance, and reduction of the ATP needed to provide energy to the H^+^ pump to regulate ion transport (Liu et al., 2014). In this study, hypoxia-stressed plants exhibited a reduction in the N, P, K, and Zn contents in their leaves, whereas Mn and Fe were increased abnormally. This may be because hypoxia decreases soil redox potential, resulting in increased amounts of soluble Fe^2+^ and Mn^2+^, thus leading to excessive uptake of these nutrients (Chen et al., 2005). Our results are in agreement with Pezeshki et al. (1999), who reported an increase in Mn and Fe contents in *Lepidium latifolium* under hypoxia stress. High uptake of Fe under hypoxia stress induces the generation of O_2_^–^ and OH^–^ by Fe-catalyzed reaction in roots, consequently leading to reduced protein biosynthesis, high lipid peroxidation, and cell death in roots (Chen et al., 2005). However, supplementation of conventional Si enhanced the N, P, K, and Zn contents in the leaves of muscadine grape plants grown under hypoxia stress. This effect was even more pronounced when SiNPs were supplied, and the plants accumulated more amounts of N, P, K, and Zn in their leaves. It is well documented that soil application of Si and SiNPs increased the nutrient supply under stress conditions (Liu and Lal, 2015). As SiNP-treated plants exhibited higher photosynthesis, it could be attributed to better nutrient uptake under hypoxia stress (Asgari et al., 2018). Besides, SiNPs also prevented plants from accumulating Mn and Fe to phytotoxic levels.

The findings of the study presented here confirmed that the exogenous application of SiNPs is more effective than conventional Si to alleviate hypoxia stress in muscadine grapes. SiNPs effectively improved muscadine plants’ growth and development under normal and low oxygen conditions. The application of SiNPs significantly mitigated hypoxia-induced oxidative damages to leaf and root tissues by upregulating the antioxidant defensive systems, resulting in efficient ROS scavenging to maintain homeostasis between ROS formation and their elimination. Furthermore, SiNPs prevented the cells from osmotic shock by accelerating the accumulation of organic osmolytes, i.e., proline and GB. Hypoxia stress reduced the bioavailability of nutrients such as N, P, K, and Zn, but SiNPs reversed this effect by improving their uptake. As mentioned above, both Mn and Fe contents were increased under hypoxia stress, but this increase was in a normal range in SiNP-treated plants. It could be an adaptation in SiNP-treated plants to prevent plant tissues from accumulation of Mn and Fe to a phytotoxic level. In this study, we analyzed nutrients in leaf tissues only; however, the effects of hypoxia stress on root physiology, nutrient uptake, and translocation will be the focus of future studies. Overall, improved hypoxia tolerance in SiNP-treated muscadine plants is attributed to the enhanced formation of compatible osmolytes (proline and GB) and enzymatic and non-enzymatic antioxidant activities, thus limiting cellular osmotic and oxidative damages. Further studies are needed to decipher the effect of SiNPs at the molecular (metabolomics and gene expression) level in alleviating oxidative and osmotic stress under low oxygen conditions caused by prolonged flooding.

## Data Availability Statement

The original contributions presented in the study are included in the article/supplementary material, further inquiries can be directed to the corresponding author/s.

## Author Contributions

ZI designed the experiment, improved the manuscript, and provided financial support for publication. AS designed the study, coordinated the experiments, provided plant materials and lab facilities, and improved the final draft. RB analyzed the data, interpreted the results, and improved the manuscript. CG provided greenhouse facilities, hydroponic systems, and associated supplies. MZ helped in setting up the experiment and replacing nutrient solution on a weekly basis, monitored pH and Ec on a daily basis, measured plant biomass, and cleaned the hydroponic units at the end of experiment. NI and NK prepared the initial draft of the manuscript. MS designed, conducted, and supervised the study, measured physiological and biochemical attributes, analyzed the data, made graphs, and edited and improved the final draft. All authors read and approved the final manuscript.

## Conflict of Interest

The authors declare that the research was conducted in the absence of any commercial or financial relationships that could be construed as a potential conflict of interest. The reviewer FG-S declared a past co-authorship with several of the authors, MS, RB, NK, and CG to the handling editor.
